# Digital Three-Dimensional Atlas of Quail Development Using High-Resolution MRI

**DOI:** 10.1100/tsw.2007.125

**Published:** 2007-05-11

**Authors:** Seth W. Ruffins, Melanie Martin, Lindsey Keough, Salina Truong, Scott E. Fraser, Russell E. Jacobs, Rusty Lansford

**Affiliations:** California Institute of Technology, Division of Biology, Biological Imaging Center, Beckman Institute, Pasadena, CA, USA

**Keywords:** anatomy, embryo, development, imaging, microscopy, avian, quail, MRI

## Abstract

We present an archetypal set of three-dimensional digital atlases of the quail embryo based on microscopic magnetic resonance imaging (μMRI). The atlases are composed of three modules: (1) images of fixed *ex ovo* quail, ranging in age from embryonic day 5 to 10 (e05 to e10); (2) a coarsely delineated anatomical atlas of the μMRI data; and (3) an organ system-based hierarchical graph linked to the anatomical delineations. The atlas is designed to be accessed using SHIVA, a free Java application. The atlas is extensible and can contain other types of information including anatomical, physiological, and functional descriptors. It can also be linked to online resources and references. This digital atlas provides a framework to place various data types, such as gene expression and cell migration data, within the normal three-dimensional anatomy of the developing quail embryo. This provides a method for the analysis and examination of the spatial relationships among the different types of information within the context of the entire embryo.

## INTRODUCTION

Embryology describes the emergence of complex anatomical forms through the growth, specification, and differentiation of the primordial germ layers. Anatomy is a primary descriptor of all organisms and represents the overt consequence of a genotype. Anatomical atlases present a precise, visual representation of the distinguishable components comprising an organism. Atlases are invaluable references for understanding the complex forms and spatial relationships of the structures that comprise organisms. Traditionally, atlases are composed of photographic plates of histological sections in conjunction with descriptive text and clarifying illustrations. However, much information is lost when presenting three-dimensional datasets in a two-dimensional book format. Ideally, atlas users can interact more dynamically with web-based embryo datasets and new data can be added continually once acquired.

To this end, we developed a magnetic resonance imaging (MRI)–acquired digital quail development atlas (QDA). Avians have been the premier, nonmammalian, vertebrate model organism for developmental studies for over 2 millennia[1]. The Japanese quail (*Coturnix coturnix japonica*) combines well-described embryology, rapid development, and ready access to the embryo, making it ideal for both molecular and imaging studies. The accessibility of the avian embryo during development permits detailed fate mapping, surgical and pharmacological perturbations, and the introduction of gene expression vectors into specific cell populations. In this way, avians are the ideal system for spatiotemporal characterization of transcription elements important during embryogenesis. The development of green fluorescent protein (GFP) reporters and advances in live microscopic imaging allow the real-time monitoring of gene activity during embryogenesis and the morphogenetic movements that shape the embryo. We are now poised to place, in time and space, patterns of gene expression, the fates of cells, and the distribution of the molecular components involved in development into the context of the embryo. The atlases described here use anatomy to provide a spatial framework for the integration of these disparate data types.

Microscopic MRI (µMRI) is a noninvasive method that permits soft tissues to be distinguished within optically opaque specimens. µMRI depends on the relaxation properties of excited hydrogen nuclei in water and lipids. When an embryo is placed in the 11.7-T static magnetic field, the hydrogen nuclei become polarized in the field[2]. During a µMRI experiments, the application of radio frequency (RF) and magnetic field gradient pulses allow three-dimensional image data to be collected directly from the intact specimen; thus, no artifacts are introduced by the physical deconstruction of the specimen. This noninvasive method offers several different sources of contrast and makes it possible to image the same specimen repeatedly over time[3,4,5].

Here we report the creation of a series of digital three-dimensional atlases of the developing quail embryo that have been anatomically annotated. We used an MRI microscope for the acquisition of volumetric data of avian embryos *ex ovo*. The data can be freely accessed at http://atlasserv.caltech.edu/Quail/Start_Quail.html. We envision that the QDA will provide a spatial scaffold for the avian research community to place domain-specific data in the context of the entire embryo.

## MATERIALS AND METHODS

### Quail Eggs

Our datasets are collected from various embryonic stages of the Japanese quail, *C. coturnix japonica*[6]. The average Coturnix egg that we used weighed ~10–11 g and measured ~33 × 27 mm. We analyzed quail embryos ranging in age from embryonic day 5 to 10 (Hamburger and Hamilton [HH] stages 26 to 37)[7] at 1-day intervals.

### Quail Embryo Preparation

Eggs were incubated between 36º and 38ºC in a rocking, humidified incubator (Petersime Incubator Co., Gettysburg, OH) and harvested from embryonic days 5 to 10. Prior to collection, eggs were cooled to room temperature. Eggs were windowed and embryos carefully cut free from the yolk and placed in a culture dish containing Ringer's Solution. Extraembryonic membranes were dissected away and embryos rinsed free of remaining yolk in Ringer's Solution. After the cessation of heart beat, the embryos were fixed in 4% paraformaldehyde at 4ºC for 24–48 h. Specimens were washed in phosphate buffered saline (PBS) and stored in PBS + 0.05% sodium azide. For imaging, excess moisture was removed from the surface of embryos by gently blotting with a Kimwipe. Embryos were then transferred to a small culture dish containing fomblin (Rusimont USA, Inc.). Aqueous solution remaining on the surface of the embryos, including ear pits and the mouth, was removed using a finely pulled glass pipette. All imaging was performed in fomblin to eliminate background signal.

## Microscopic MRI

High-resolution µMRI was performed at 4°C using an 11.7-T, vertical bore (89 mm) Bruker DMX500 Avance spectrometer (Bruker Instruments, Billerica, MA). We used a Micro 2.5–shielded gradient set (max 100 Gauss/cm gradient strength) with Bruker 20-mm diameter RF coil and low-noise preamplifier. Images used to create the atlas were recorded using three-dimensional rapid acquisition with relaxation enhancement (RARE) protocols[8], with a data matrix of 256 × 256 × 256 points for the e09 and younger embryos and 512 × 256 × 256 for the e10 embryos. Typical spatial resolution was 34–90 mm per voxel, depending on the size of the embryo. The imaging parameters are shown in Table 1. Note that e06 and e08 datasets have multiple echo times.

**TABLE 1**

| Embryonic Age | Voxel Size (µm) | TR (msec) | TE (msec) | Rare Factor | Scans Averaged | Acquisition Time (HH:MM:SS) |
|---|---|---|---|---|---|---|
| e05 | 18 × 34 × 18 µm | 1425 | 16.2 | 8 | 4 | 14:04:35 |
| e06 | 39 $\mu m^3$ | 2100 | 20 | 8 | 4 | 20:28:48 |
| e06 | 39 $\mu m^3$ | 2000 | 25 | 8 | 3 | 14:56:00 |
| e07 | 45 $\mu m^3$ | 1000 | 25 | 8 | 3 | 8:06:24 |
| e08 | 57 $\mu m^3$ | 1400 | 30.2 | 8 | 3 | 11:06:12 |
| e08 | 57 $\mu m^3$ | 1400 | 40 | 8 | 3 | 11:36:19 |
| e08 | 57 $\mu m^3$ | 1400 | 14 | 8 | 3 | 10:16:26 |
| e09 | 70 $\mu m^3$ | 1300 | 13.6 | 8 | 2 | 6:22:50 |
| e10 | 45 × 90 × 45 µm | 1000 | 14.1 | 8 | 2 | 5:01:56 |

See Dhenain et al.[12] and references therein for further discussion of MRI.

For imaging, embryos were placed in custom-machined Teflon holders appropriate for the size of the specimens, and MRI sample tubes of either 10- or 20-mm internal diameter. The early embryos were secured to the Teflon holder with a small globule of 112 high-performance lubricant (Dow Corning). For early stages (e05 and e06), the buoyancy of the embryo in fomblin was exploited to position the embryo in the sample tube. Larger specimens were secured by wrapping them in Teflon tape. In these cases, great care was taken to maintain the embryo’s posture. Gently tapping and rocking the sample tube coaxed bubbles free that were trapped by the holders.

## Software Resources

We used the following commercial software: Amira (Mercury Computer Systems, www.tgs.com) and VGStudio (Volume Graphics, www.volumegraphics.com). We used the following free software: SHIVA (Synchronized Histological Image Viewing Architecture) and BrainSuite2 (www.loni.ucla.edu/Software/ and ImageJ (http://rsb.info.nih.gov/ij/). The ImageJ plugins used were: Analyzer Reader/Writer, AmiraMesh Reader/Writer, and ShowAmiraSurface.

## Delineating Anatomical Structures

Identified anatomy was manually labeled in each of the three orthogonal data collection planes using the segmentation tools available in Amira visualization software (Mercury Computer Systems). A labeling session involves the creation of an 8-bit image file (label file) with the same dimensions as the µMRI data volume being segmented. Essentially, each identified anatomical structure is "painted" into the label file using the µMRI dataset as the template. Each painted region is assigned a label that consists of a unique numerical identifier, the structure's name, and a color code.

Nomenclature and identification of structures were based principally on previously published avian atlases[6,7,9,10]. In most instances, the boundaries between tissues were obvious. However, for some structures, the boundaries were not easily identifiable. In these cases, as is commonly done for atlas annotation purposes[11], boundaries were placed according to the following criteria: (1) three-dimensional morphology (organization of the structure as visible on adjacent and orthogonal slices), (2) position of surrounding tissues, and (3) comparison with position of the structure in existing histological atlases[9,12]. Only structures and portions of structures that could be definitively identified were labeled.

Three-dimensional surface models of labeled anatomy were generated using a generalized marching cube algorithm. The resulting surfaces were smoothed and appropriately decimated to approximate the delineated anatomy closely. Volumetric models were extracted from the µMRI data by using the label image as masks of anatomical components.

### Data Preparation for Interactive Atlas

We use SHIVA software to view the annotated atlas. SHIVA was developed at the Laboratory of Neuro Imaging at UCLA to provide a facile method for viewing multiple, overlaid image datasets. In order to use the atlas data with SHIVA, all image files were converted to Analyze Image format as was the image portion of Amira label files. The text portion of the Amira label file, which contains the label names, was parsed into a LONI label file (.lbl) and "integrated label file" (.ilf). Simple organ system hierarchies for each stage were assembled in SHIVA using the "BrainGraph" editing tool.

## RESULTS

The QDAs are each composed of three components. First, they contain unprocessed µMRI volumes of fixed embryos aged 5–10 days. Image volumes can be viewed as a series of sections or volume rendered with appropriate software (Fig. 1). Second, the atlases contain a coarsely delineated anatomy of e05, e06, e07, e08, e09, and e10 quail embryos. Third, anatomy has been grouped by functional system and displayed in a hierarchical graph. The atlas can be viewed with SHIVA. Finally, we have made available a set of three-dimensional surface models of the e05, e06, e07, e08, e09, and e10 quail embryo anatomy. With appropriate software, the whole embryo or specific organs (e.g., heart, liver, kidneys, etc.) can be displayed as three-dimensional objects.

### µMRI and Raw Image Presentation

One to three MRI datasets were collected for each embryo using different T2 echo times to optimize the contrast of different structures (Fig. 2 and Table 1). The relaxation time varies between tissues due to the physical properties and the chemical environment of water[2]. The data were converted from 16 to 8 bit to conserve computer memory, to improve processing speed, and to facilitate data transfer. The converted 8-bit images are visually indistinguishable from the 16-bit source data. Image volumes are delivered via the Internet in Analyze Image format, a standard image format that is readable by several visualization applications including SHIVA. Image volumes were sectioned along user-defined planes using SHIVA (Fig. 3). Other applications, such as Amira, VGstudio, and ImageJ, allow the user to display volume renderings from any point of view.

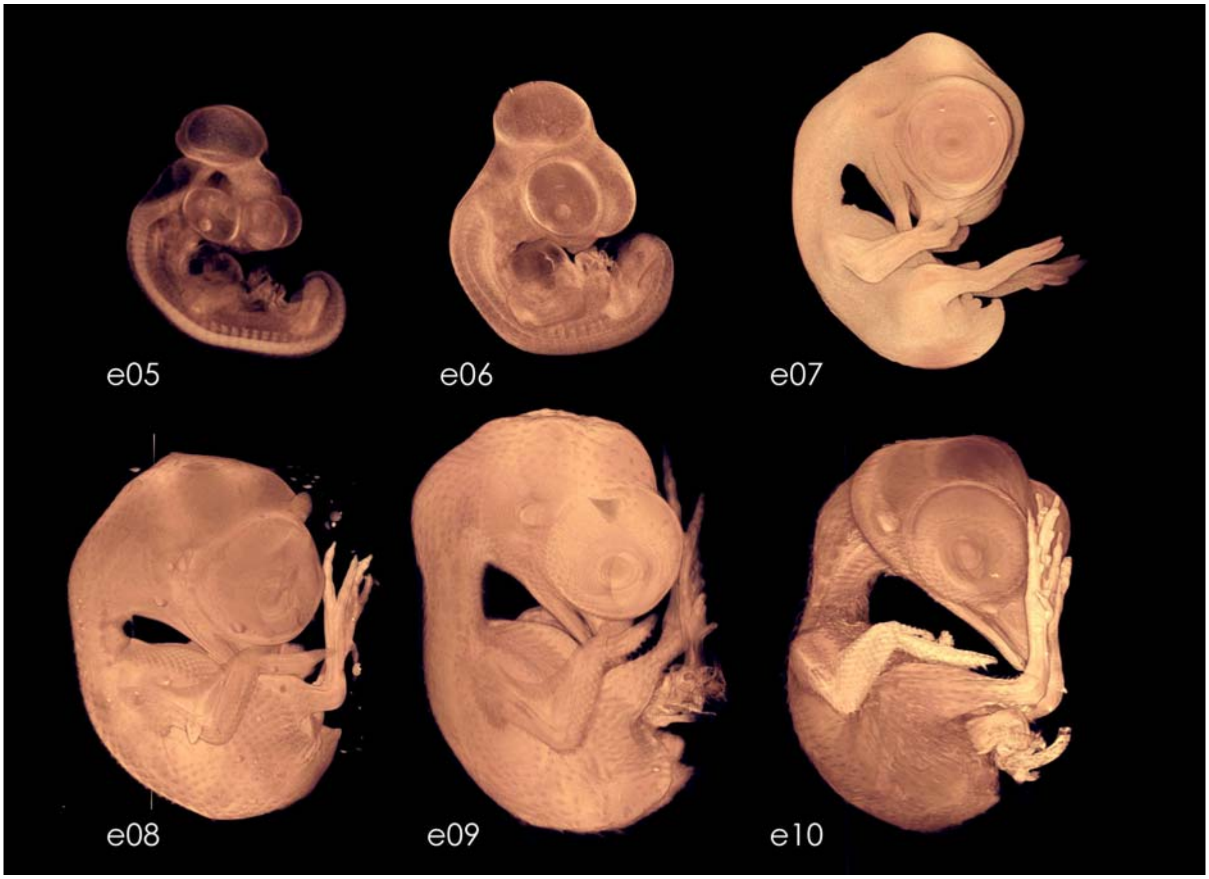

**FIGURE 1**. Volume renderings of the µMRI datasets used in the QDA for quail embryos representing days e05–e10 are shown.

## Image Segmentation/Anatomical Labeling

The MRI slices were used as the template for labeling major anatomical structures. Numerous identifiable structures were "painted" and annotated, however, not all recognizable structures have yet been labeled. Raw image data were primarily used to delineate anatomy, however, three-dimensional Gaussian filtering was used to aid in deciding certain structure boundaries (e.g., to identify the boundaries of the neck vertebrae in e08). We used published atlases to confirm identification of the structures[6,7,10].

We have identified and annotated numerous major anatomical structures for each embryo. There are still many other anatomical structures that could be identified and labeled in the QDAs by specialists in anatomy and developmental biologists with the specific expertise. This is, in part, why we have made the various QDAs freely accessible and downloadable.

Using SHIVA's BrainGraph Editor, we grouped anatomical labels into defined organ systems (Fig. 4). For instance, the nervous system grouping includes the spinal cord and all identified brain structures. The skeletal system grouping includes all identified bones including the scapula, vertebrae, and femurs, to name a few.

SHIVA also permits interactive browsing and navigation of the atlases. The current location and anatomical structure name is displayed as the cursor is passed over the image (Fig. 4). For example, using the computer mouse to "Ctrl+click" on a particular anatomical image highlights and centers the associated label in the tree graph viewer. Conversely, selecting an associated label in the tree graph viewer places the cursor on the proper anatomical image in the image view panes. This permits the user to access relationships between anatomical structures quickly.

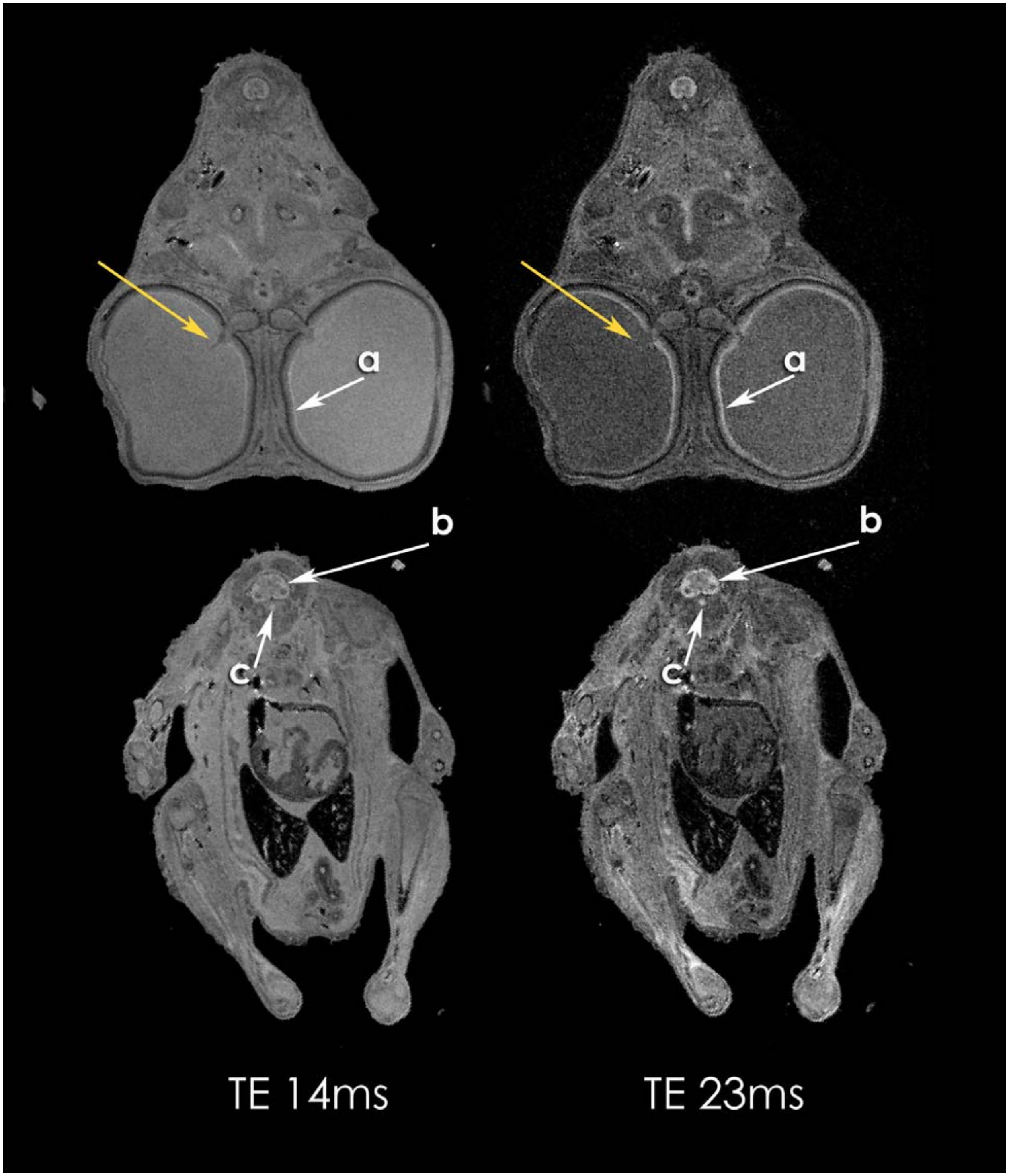

**FIGURE 2.** Multiple MRI collection schemes can be exploited for anatomical labeling. Since the relaxation time of water is dependent on its chemical and physical environment, collecting multiple echoes reveals different structures. Short relaxation times produce a low noise image with good contrast between many tissues. In the TE 14-ms image (left), the pecten/optic nerve is visible within the vitreous chamber (yellow arrow), but invisible in the TE 23-ms image (right). Longer relaxation times produce higher noise images, but also provide higher and distinct contrast. Although the TE 23-ms image is much noisier, white matter in the retinal neural layer (a), spinal cord (b), and notochord (c) are very bright when compared with the TE 14-ms image.

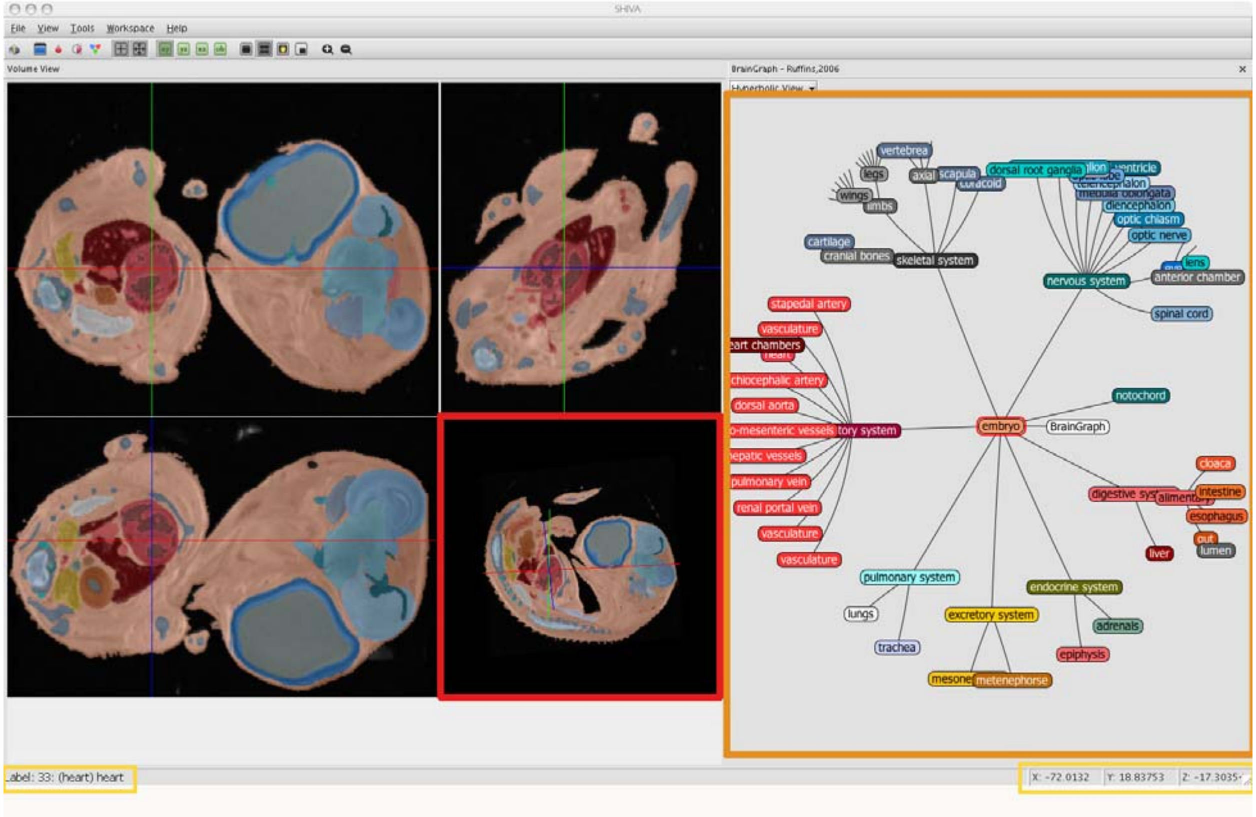

**FIGURE 3**. SHIVA Atlas viewer. The atlases are designed to work with SHIVA, a free Java application (UCLA-LONI; see text for download). The main SHIVA window is the "Volume View", which displays orthogonal sections through the image volume and an arbitrary section plane (highlighted in red). Labeled anatomy is displayed as a color overlay on the MRI. As the user cursors over the embryo image in the Volume View, the current structure name and cursor location are displayed in the lower left and right data fields, respectively (highlighted in yellow). The "BrainGraph" window (highlighted in orange) displays a tree graph of our simple organ system hierarchy. The anatomical labels are represented as colored nodes in the graph. Shown here is the hyperbolic view of our hierarchy. Selecting a node in the hyperbolic graph in the right pane places the cursor on the selected anatomy. (Ctrl+click) on anatomy in the volume viewer centers the associated node in the graph view.

## Three-Dimensional Reconstruction and Rendering of Anatomy

We generated surface models for each labeled anatomical structure using Amira's Generalized Marching Cube algorithm. Each surface is a mesh composed of a set of triangular patches defining the surface. The number and size of the triangles defines how closely the surface approximates the boundaries of the volumetric labels. High-density meshes are required for small and highly convoluted structures, such as blood vessels, while low-density meshes are adequate for large structures, such as the liver (Fig. 4). For the purpose of visualization, surfaces are smoothed to remove "stair-steps", the jagged edges seen in the label volumes. Surface models also provide a means for computing the surface area and the enclosed volume of a labeled anatomical structure within the embryos.

We also used the label images as masks to extract anatomical images from the MRI data volumes directly. For example, a label is used to mask a region in an MRI data volume where the image will be preserved. Outside of the masked region, the image intensity is set to black. Anatomical image extraction is a direct method for isolating MRI data of individually labeled anatomy.

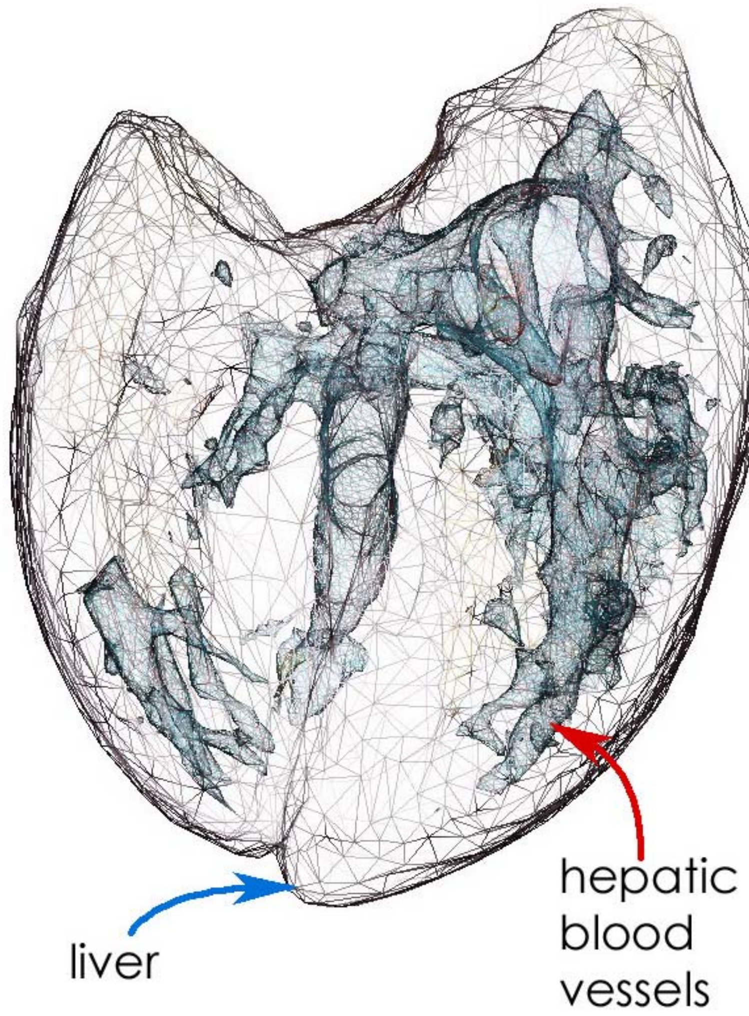

**FIGURE 4**. Wireframe rendering of a surface model of the liver and its hepatic blood vessels of e08 quail embryo. Surface models are composed of a mesh of triangular patches. Mesh densities need to be higher to approximate the shape of fine structures, such as blood vessels, more closely. Lower-density meshes are adequate to approximate large structures, such as the liver.

Anatomical volumes and surfaces provide qualitatively different types of visualizations of anatomy (Figs. 5A and 5D). Surfaces models can be rendered very quickly and display unambiguous anatomical boundaries and morphology. For example, feather buds are clearly visible on the embryo. However, surfaces lack internal information and display only structures that have been labeled (Fig, 5D).

Volume models generally render less quickly than surface models, but contain information within the boundaries of the labels. Structures that have not been explicitly labeled are visible in volume renderings. Notice in Fig. 5D that muscle groups are visible in the leg. However, volume renderings can have visually ambiguous boundaries, such as the tail in Figs. 5B and 5C. The loss of visibility is due to the application of transparency to low signal (dim areas) to remove noise and to reveal internal structures. Consequently, regions with low signal become invisible. In either case, the ability to render the embryo in three-dimensions allows the user to examine anatomy within the context of the entire embryo.

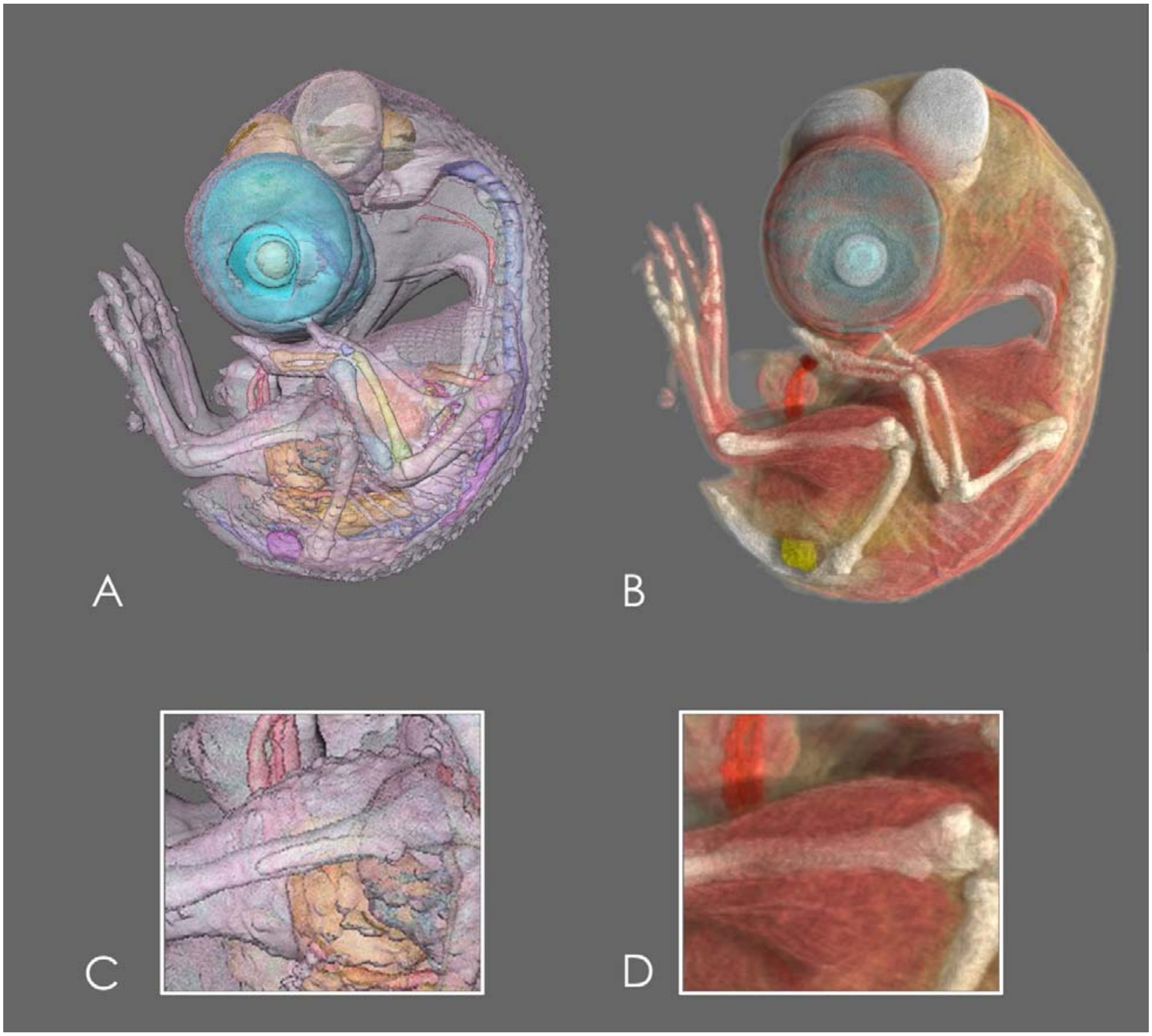

**FIGURE 5**. Three-dimensional renderings of e08 quail developmental atlas. (A) Shows a surface rendering of all labeled anatomical structures in the e08 atlas. In this rendering, the transparent surfaces allow all structures to be displayed. Surface colors are derived from the labels. (B) Displays a volume rendering of extracted anatomy from the e08 atlas. Anatomy was extracted in organ system groups. For example, the entire nervous system was extracted as a single group. Color and transparency attributes were assigned to each group to help to visually distinguish organ systems. C and D are details from A and B, respectively. (C) Shows that surface renderings can display boundaries unambiguously. Note that feather buds are visible in A and C, but not B or D. Surfaces contain no internal information. (D) The embryo surface and the tibiotarsus can be seen with no intervening material. In B and D, the volume renderings show that tissue lies between the surface and the bone, and although muscle has not been labeled explicitly, muscle compartments separated by bright regions of fascia membranes are visible.

## DISCUSSION

We present a series of embryonic quail atlases ranging in age from embryonic day 5 through 10 (Fig. 1). Unlike traditional atlases, which are based on histological sections, these atlases are built from µMRI, a nondestructive imaging modality that preserves the native morphology of tissues. Because MRI derives contrast from the paramagnetic properties of water and its chemical and physiological environment, not the optical properties of the tissues, minimal specimen preparation is necessary. Dehydration and sectioning artifacts are nonexistent in our specimens because the entire volume of the embryos is imaged at once. Additionally, MRI produces registered image volumes, making three-dimensional reconstruction trivial. While the resolution of µMRI is an order of magnitude lower than optical methods, it is sufficient for imaging organ systems (voxel sizes can be as small as 25 $\mu m^3$)[13]. At this resolution, these atlases represent some of the most spatially and morphologically accurate anatomical images of avian development to date. Continued hardware development that permits higher resolution analysis will allow µMRI data for smaller, earlier stages to be collected in the future. Nondestructive optical methods, such as optical projection tomography and confocal microscope imaging, are currently used to extend the atlas datasets to earlier embryonic stages. Some of these datasets collected with different imaging modality are currently being linked and integrated with the QDA µMRI datasets.

Another departure from traditional atlases is volumetric labeling of anatomy. Typically, anatomy is labeled using simple pointers and index numbers. Here, each voxel associated with an identified piece of anatomy is assigned a name, a numerical tag, and a color. Tissues boundaries are defined resulting in volumes that represent entire structures. The morphology of individual structures can be visualized as surfaces and/or volumes (Fig. 5). Additionally, MRI allows quantitative morphological measurements of unperturbed anatomy to be made.

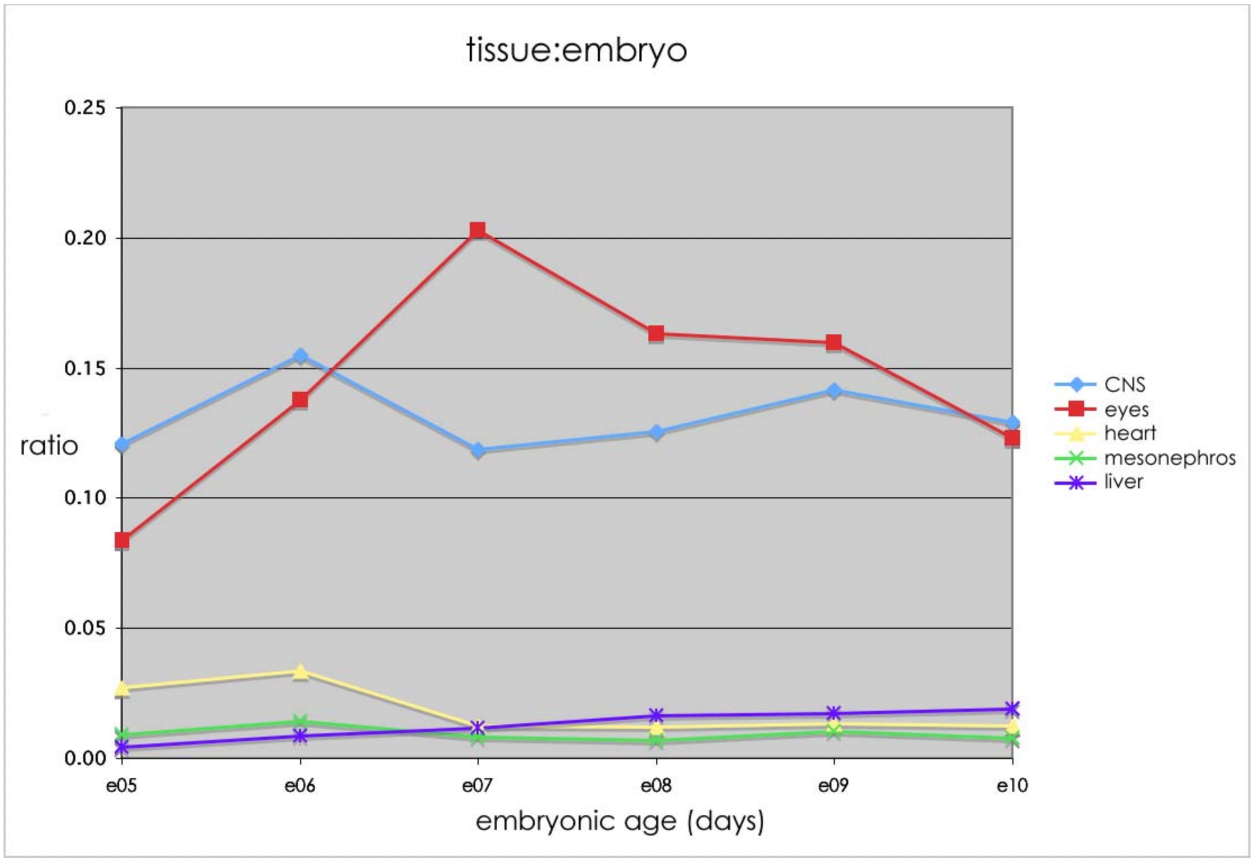

**Figure 6.** An example of volumetric analysis. The ratio between the volume of anatomical structures and the embryo shows the relative rates of tissue expansion between e05 and e10. Volumetric analysis could reveal subtle changes as the result of experimental manipulation.

Each of the atlases is constructed from a representative individual for each day of development from e05 through e10. Embryos to be imaged were assessed for health and “normal” appearance. Egg size was not considered during the harvesting of embryos. Although the anatomic morphology is virtually unperturbed, the normal variability between individuals precludes a straightforward analysis of embryonic and organ growth between these six embryos (Table 2). However, as an example of how volumetric MRI data can be used for morphometric measurements, we normalized the variability between specimens by using the tissue:embryo ratio for each specimen. For the embryos used for the construction of these atlases, the ratio between the embryo and the heart, liver, mesonephros, and CNS remains constant between e05 and e10. The eyes seem to grow rapidly between e05 and e07, then grow more slowly between e07 and e10 (Fig. 6).

The granularity of embryonic staging is dependent on the criteria for staging. For example, H-H staging is based on the external anatomical features. However, a gene expression staging system could provide a method to stage embryos at very short intervals. The staging system used for the compilation of these atlases proved to be a convenient staging method and clearly shows the dramatic morphological changes that occur at daily intervals.

**TABLE 2**

| **Embryonic day** | **e05** | **e06** | **e07** | **e08** | **e09** | **e10** |
|---|---|---|---|---|---|---|
| Volume (µm$^3$) | | | | | | |
| Liver | 0.83 | 0.97 | 4.42 | 21.02 | 16.14 | 28.66 |
| lungs | 0.41 | | 1.70 | 11.17 | 10.94 | 17.39 |
| CNS | 22.28 | 17.19 | 44.39 | 160.70 | 132.77 | 194.98 |
| eyes | 15.38 | 15.29 | 75.96 | 209.00 | 149.82 | 185.86 |
| heart | 5.00 | 3.73 | 4.74 | 15.60 | 12.43 | 19.05 |
| mesonephros | 1.69 | 1.58 | 3.06 | 9.04 | 9.66 | 11.47 |
| Total Embryo volume (µm$^3$) | 184 | 111 | 374 | 1280 | 938 | 1511 |
| Ratio (organ/embryo) | | | | | | |
| liver | 0.00453 | 0.00870 | 0.01183 | 0.01642 | 0.01721 | 0.01897 |
| lungs | 0.00222 | 0.00000 | 0.00455 | 0.00873 | 0.01166 | 0.01151 |
| CNS | 0.12109 | 0.15486 | 0.11869 | 0.12555 | 0.14155 | 0.12904 |
| eyes | 0.08359 | 0.13775 | 0.20310 | 0.16328 | 0.15972 | 0.12300 |
| heart | 0.02717 | 0.03357 | 0.01268 | 0.01219 | 0.01326 | 0.01261 |
| mesonephros | 0.00919 | 0.01423 | 0.00818 | 0.00706 | 0.01030 | 0.00759 |

## Entering Other Types of Information into the Model

An important aspect of digital developmental atlases is the ability to update continuously and extend the information content associated with the embryo. As the events of development reside within the embryonic soma, a model of the embryo provides a logical and unifying framework for placing and accessing the vast quantities and disparate data types used to describe development. Although the atlas is constructed using µMRI as the underlying image source, data from other imaging modalities can be readily mapped into the atlas. For instance, we have begun to integrate gene expression data from transgenic quail expression GFP under the transcriptional control of tissue-specific promoters into the QDA. We plan to also integrate the QDA-acquired data for cell migration, cell fate, cell division, and extracellular matrix (ECM) distribution information, to name a few. Once the embryonic framework is populated with spatially mapped data, correlations between cell lineage, migration routes, ECM, adhesion molecules, and gene expression can be examined and explored using pattern-determining software.

The simple organ system hierarchy associated with the atlases is but one ontology for organizing anatomical information. We are currently constructing a lineage hierarchy that will link structures between stages, providing continuity between stages. The lineage hierarchy will unify embryonic space and time so the history (forward and backward) of specific tissues can be navigated.

### Education

For centuries, the compelling images found in anatomical references have been invaluable for learning how the intricate structures comprising an organism are arranged. These images have inspired wonder and curiosity, and spawned further investigation of the workings of living things. The three-dimensional atlases presented here have the ability to be explored in a three dimensional manner using the WWW, which is profoundly different from traditional sources. The embryo image volumes can be dynamically “handled” (rotated and sliced) in a manner that actual embryos cannot. Multiple organ systems can be extracted and viewed in any combination. A modified version of the QDA is currently being developed as museum exhibits in order to educate a broader audience.

### Invitation to Label and Annotate

Manual labeling of the embryonic anatomy is a labor-intensive task requiring specific knowledge. We appreciate that the current incarnation of our atlas is incomplete, but acknowledge that it serves as a foundation for continued anatomical labeling and data integration from other research labs worldwide. As the atlas is infinitely extensible and updateable, we invite the avian biology community to aid in the labeling and annotation of the QDA. The growth of Wikipedia has shown that pooled knowledge can produce an incredibly useful resource. A shared effort among the experts in this community could result in unsurpassed and highly detailed anatomical maps of the developing avian embryo. We have taken efforts to ensure that the QDA is freely available to all researchers. A “how to” guide to “atlasing” is available on the QDA website.

## ACKNOWLEDGMENTS

This work was funded, in part, by the NIH NCRR (R21HD047347-01A2), the NIH NCRR (U24 RR021760) BIRN, and the NIH NHGRI Center for Excellence in Genomic Science (P50 HG004071).
